# Syphilis and Marriage

**Published:** 1881-03

**Authors:** 


Syphilis and Marriage. Lectures delivered at the St. Louis Hospital, Paris. By Alfred Fournier, Prof. a la Faculte de Medecin de Paris, etc. Translated by P. Albert Merroid, M.D., Physician to the Skin and Venereal Department, New York Dispensary, etc. D. Appleton & Co., New York. 1881. Price $2.00, cloth.

This is a very important subject, and is fully treated in all its bearings by the author. The foolish idea that has very recently been ventilated as to the benignancy of syphilis, is very erroneous and deceptive. The terrible havoc that this protean disease is exerting among all classes should be fully understood and appreciated, and means earnestly sought that are calculated to stay its progress. The work before us discusses, in all its bearings, the influence of the disease in one of the most important social relations.

